# Pharmacological Inhibition of MDM2 Induces Apoptosis in p53-Mutated Triple-Negative Breast Cancer

**DOI:** 10.3390/ijms26031078

**Published:** 2025-01-26

**Authors:** Jasmin Linh On, Sahel Ghaderi, Carina Rittmann, Greta Hoffmann, Franziska Gier, Vitalij Woloschin, Jia-Wey Tu, Sanil Bhatia, Andrea Kulik, Dieter Niederacher, Hans Neubauer, Thomas Kurz, Tanja Fehm, Knud Esser

**Affiliations:** 1 Department of Obstetrics and Gynecology, Medical Faculty and University Hospital, Heinrich Heine University Düsseldorf, Moorenstr. 5, 40225 Düsseldorf, Germany; jasmin.on@hhu.de (J.L.O.);; 2 Center for Integrated Oncology (CIO), Aachen, Bonn, Cologne, Düsseldorf (ABCD), Kerpener Str. 62, 50937 Cologne, Germany; 3 Institute of Pharmaceutical and Medicinal Chemistry, Faculty of Mathematics and Natural Sciences, Heinrich Heine University Düsseldorf, Universitätsstr. 1, 40225 Düsseldorf, Germany; 4 Department of Pediatric Oncology, Hematology and Clinical Immunology, Medical Faculty and University Hospital, Heinrich Heine University Düsseldorf, Moorenstr. 5, 40225 Düsseldorf, Germany

**Keywords:** triple-negative breast cancer, apoptosis, MDM2 inhibitor, p53 mutation

## Abstract

Triple-negative breast cancer (TNBC) represents the most aggressive breast carcinoma subtype lacking efficient therapeutic options. A promising approach in cancer treatment is the pharmacological inhibition of murine double minute 2 (MDM2)-p53 interaction inducing apoptosis in p53 wild-type tumors. However, the role of MDM2 in TNBC with primarily mutant p53 is not well understood. We here selected the clinical-stage MDM2 inhibitors Idasanutlin and Milademetan and investigated their anti-tumoral effects in TNBC. When we analyzed anti-tumor activity in the TNBC cell lines MDA-MB-231, MDA-MB-436, and MDA-MB-468, cellular viability was efficiently reduced, with half maximal inhibitory concentration (IC_50_) values ranging between 2.00 and 7.62 µM being up to 11-fold lower compared to the well-characterized non-clinical-stage MDM2 inhibitor Nutlin-3a. Furthermore, caspase-3/7 activity was efficiently induced. Importantly, the IC_50_ values for MDM2 inhibition were equally observed in HCT116 *p53^+/+^* or HCT116 *p53^−/−^* cells. Finally, the IC_50_ was significantly higher in non-malignant MCF-10A cells than in TNBC cells. Taken together, Idasanutlin and Milademetan show a potent anti-tumor activity in TNBC cell culture models by efficiently inducing tumor cell death via apoptosis. This effect was observed despite an inactivating p53 mutation and was apparently independent of p53 expression. Our data suggest that MDM2 is a promising target in TNBC and clinical-stage MDM2 inhibitors should be further evaluated for their potential therapeutic application.

## 1. Introduction

Triple-negative breast cancer (TNBC) accounts for 15–20% of all breast cancer subtypes and represents the most aggressive subtype. Due to the lack of cellular targets well known in other breast cancer subtypes, namely estrogen, progesterone, and human epidermal growth factor 2 receptors, therapeutic options are limited. Additionally, the current TNBC treatment regimens are broadly based on (neo-)adjuvant chemotherapies associated with significant side effects and often leading to high chemoresistance [1]. Although with immunotherapeutic agents and poly-adenosine diphosphate-ribose polymerase (PARP) inhibitors more targeted therapeutic options are available [1], these drugs are often limited to distinct TNBC subgroups and regularly lead to therapy resistance development early after therapy initiation [2,3]. Therefore, novel therapeutic strategies are urgently needed. However, the identification of new cellular targets in TNBC remains challenging.

To date, different cellular processes have been described to be dysregulated in TNBC, offering novel concepts for optimizing personalized treatment. Current strategies include studies with epidermal growth factor (EGF) receptor antagonists, phosphoinositide 3-kinase (PIK3CA), proteinkinase B (AKT), and mechanistic target of rapamycin (mTOR) inhibitors, as well as inhibitors of Wnt/β-Catenin, NOTCH, and the transforming growth factor (TGF) receptor or the RAS/RAF/MEK/ERK signaling pathway [4,5,6]. Interestingly, the p53-murine double minute 2 (MDM2) pathway, despite playing an essential role in preventing tumor development and regulating cellular processes [7], has rarely been investigated in TNBC. In fact, p53 mutation is the most common mutation among all human cancers (~50%) [7] and occurs in 30% of all breast cancer cases (80% in TNBC) [8]. Apart from p53 inactivation by gene loss-of-function mutations, p53 is inhibited by various mechanisms, with overexpression of MDM2 representing an effective principle for negatively regulating p53 function [9]. The overexpression of MDM2 is frequently found in soft tissues (20%) [9] and mainly leads to a bad prognosis [10,11]. In breast cancer, MDM2 overexpression occurs in approximately 6% of cases [9], with this increasing to 14% in the TNBC subtype [8,12].

Pharmacological inhibitors of MDM2 specifically address the MDM2-p53 protein–protein interaction, leading to the activation of p53 and the induction of cellular apoptosis or cell cycle arrest. MDM2 inhibitors are known to target cancer cell viability in p53 wild-type cancers without genotoxic effects [13]. However, little is known about their anti-tumor activity in p53-mutant cancers so far. Recent studies in colon cancer cells point at a significant anti-tumor activity of MDM2 inhibitors also in p53-mutated cells [14,15]. In fact, Nutlin-3a has been shown to induce apoptosis in TNBC at higher micromolar concentrations [16,17]. Furthermore, synergistic applications to chemotherapeutics reveal that pharmacological targeting of MDM2 might also be beneficial to current treatment regimes in p53-mutated TNBC [16,17]. However, potent inhibitors of MDM2 activity now being tested in clinical trials of p53 wild-type cancers have not yet been studied for anti-tumor activity in TNBC. Therefore, we investigated whether TNBCs, which mostly carry inactivating p53 mutations [8], could be treated efficiently using the newly described MDM2-targeting highly potent clinical-stage inhibitors Idasanutlin and Milademetan. Both inhibitors exhibit high binding activity against MDM2, with IC_50_ values of 6 nM and 5.57 nM [18,19], respectively, being 15.8- and 14.7-fold higher compared to the non-clinical-stage MDM2 inhibitor Nutlin-3a (IC_50_ = 88 nM) [20,21], whose high target specificity has been widely investigated in substantial cellular and non-cellular studies [20,22,23,24] and was chosen for reference in our studies.

We here assessed the effect of potent clinical-stage MDM2 inhibition on cellular viability in three different TNBC cell lines carrying p53-inactivating mutations (MDA-MB-231: R280K; MDA-MB-436: E204fsX45; and MDA-MB-468: R273H [25]) and further characterized the mechanisms of cell death. Furthermore, we investigated the p53-independent mode of action of MDM2 inhibition by assessing cellular viability regardless of p53 expression. Finally, to estimate potential side effects in healthy tissues and to evaluate a feasible pharmaceutic window in future medical applications, we studied the cellular viability in non-malignant cells treated with MDM2 inhibitors.

## 2. Results

### 2.1. Clinical-Stage Inhibitors of MDM2 Potently Reduce Cell Viability in p53-Mutated TNBC Cell Lines

Since previous studies suggest a significant anti-tumor activity for MDM2 inhibitors in p53-mutated cancer cells [14] and the non-clinical-stage inhibitor Nutlin-3a has been shown to induce apoptosis in TNBC at higher micromolar concentrations [16,17], we aimed to address if novel clinical-stage MDM2 inhibitors that possess an increased pharmacological potency over Nutlin-3a efficiently [26] target cell viability in TNBC.

**Figure 1:**
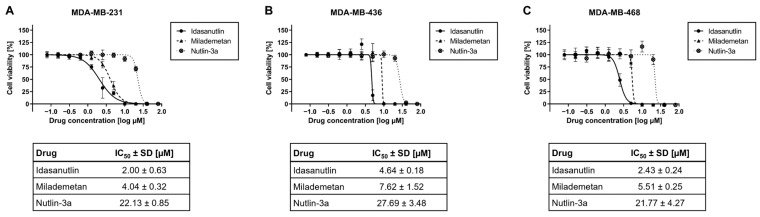
Analysis of cell viability in TNBC cell lines after incubation with murine double minute 2 (MDM2) inhibitors. MDA-MB-231 (**A**), MDA-MB-436 (**B**), and MDA-MB-468 (**C**) were seeded and incubated for 24 h before treatment. Idasanutlin, Milademetan, or Nutlin-3a were added at the displayed concentrations and incubated for 72 h. Treatment was repeated for another 72 h. Subsequently, cell viability was determined using the CellTiter-Glo assay. The graphs show the mean ± SD n ≥ 3. Tables below the graphs contains the summarized IC_50_ values ± SD.

We therefore decided to investigate the anti-tumor activity of the potent MDM2 inhibitors Idasanutlin and Milademetan, which are currently being tested in clinical trials of p53 wild-type tumors [13], in the p53-mutated TNBC cell lines MDA-MB-231, MDA-MB-436, and MDA-MB-468. Cells were treated with different concentrations of the selected MDM2 inhibitors or the reference compound Nutlin-3a, a well-characterized non-clinical-stage inhibitor of MDM2-p53 interaction [20]. After performing cell viability assays, inhibitory dose–response curves were generated and IC_50_ values were calculated. All MDM2 inhibitors significantly reduced the cell viability in a dose-dependent manner in all TNBC cell lines (Figure 1). The IC_50_ values of Idasanutlin in MDA-MB-231, MDA-MB-436, and MDA-MB-468 were 2.00 ± 0.63 µM, 4.64 ± 0.18 µM, and 2.43 ± 0.24 µM, respectively. The IC_50_ values of Milademetan in MDA-MB-231, MDA-MB-436, and MDA-MB-468 were 4.04 ± 0.32 µM, 7.62 ± 1.52 µM, and 5.51 ± 0.25 µM, respectively, while the corresponding IC_50_ of Nutlin-3a were 22.13 ± 0.85 µM, 27.69 ± 3.48 µM, and 21.77 ± 4.27 µM, residing in a markedly higher range. In contrast to the tested MDM2 inhibitors, dimethyl sulfoxide (DMSO) alone induced no cell death in the TNBC cell lines at the tested concentrations (Figure S1).

These data illustrate that MDM2 inhibitors efficiently target the cancer cell viability of p53-mutated TNBC cells. Furthermore, Idasanutlin and Milademetan possess pharmacological potencies that are at least approximately sixfold superior to the non-clinical-stage reference compound Nutlin-3a.

### 2.2. MDM2 Inhibitors Induce Increased Caspase-3/7 Activity in MDA-MB-231 Cells

p53 has been widely shown to regulate cellular apoptosis induction and mediate cellular programmed cell death [7]. Furthermore, activation of wild-type p53 by MDM2 inhibitors induces apoptosis [13]. We therefore investigated the caspase-3/7 activity in p53-mutated MDA-MB-231 cells.

**Figure 2:**
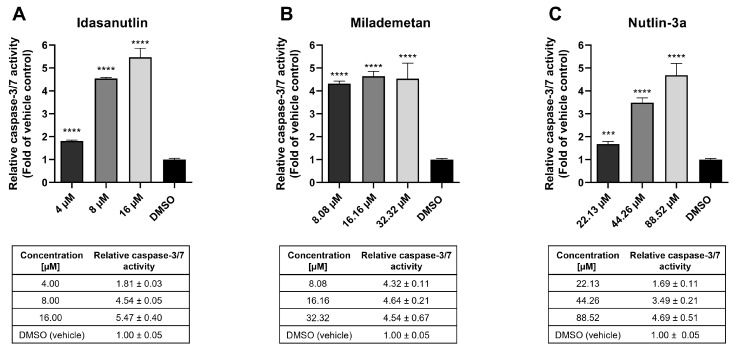
Analysis of apoptosis induction in MDA-MB-231 cells after incubation with murine double minute 2 (MDM2) inhibitors. MDA-MB-231 cells were seeded and incubated for 24 h before treatment. Cells were treated with toxic concentrations of MDM2 inhibitors (up to an 8-fold increase of the corresponding IC_50_ concentration) and incubated for 24 h. Idasanutlin with concentrations between 4 and 16 µM (**A**), Milademetan with concentrations between 8.08 and 32.32 µM (**B**), and Nutlin-3a with concentrations between 22.13 and 88.52 µM (**C**). Subsequently, caspase-3/7 activity was evaluated using the Caspase-Glo 3/7 assay. Dimethyl sulfoxide (DMSO) in concentrations corresponding to highest compound concentrations was used as control. The graphs show the mean ± SD n ≥ 3. Tables shown below the graphs contain the mean caspase-3/7 activity ± SD. *** *p* < 0.001, **** *p* < 0.0001.

MDA-MB-231 cells were treated with toxic concentrations of Idasanutlin, Milademetan, and Nutlin-3a (up to an 8-fold increase of the corresponding IC_50_ concentration). As shown in Figure 2, the caspase-3/7 activity of cells treated with 4 to 16 µM Idasanutlin was 1.81 ± 0.03-, 4.54 ± 0.05-, and 5.47 ± 0.4-fold compared to vehicle control (DMSO). Similarly, for Milademetan, caspase-3/7 activity was 4.32 ± 0.11-, 4.64 ± 0.21-, and 4.54 ± 0.67-fold higher compared to vehicle control when treated with 8.08 to 32.32 µM of the compound. For Nutlin-3a, serving as reference, 1.69 ± 0.11-, 3.49 ± 0.21-, and 4.69 ± 0.51-fold increases in caspase-3/7 activity compared to vehicle control were detected when cells were treated with 22.13 to 88.52 µM of the compound.

These data illustrate a significant increase in caspase-3/7 activity in MDA-MB-231 cells treated with Idasanutlin, Milademetan, and Nutlin-3a, revealing an efficient induction of apoptosis in these cells.

### 2.3. Pharmacological MDM2 Inhibition Induces Cell Death Independent of Cellular p53 Expression

**Figure 3:**
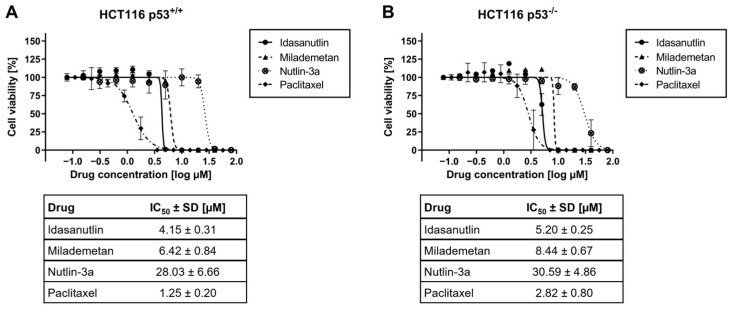
Effect of murine double minute 2 (MDM2) inhibitor Nutlin-3a on cell viability in HCT116 *p53^+/+^* and HCT116 *p53^−/−^* cells. HCT116 *p53^+/+^* (**A**) and HCT116 *p53^−/−^* (**B**) cells were seeded and incubated for 24 h before treatment. Nutlin-3a or Paclitaxel was added at the displayed concentrations and incubated for 72 h. Treatment was repeated for another 72 h. Subsequently, cell viability was determined via the CellTiter-Glo assay. The graphs show the mean ± SD n ≥ 3. Tables listed below the graphs contain the summarized IC_50_ values ± SD.

As our investigation illustrated a pronounced anti-tumoral effect of Idasanutlin, Milademetan, and Nutlin-3a in the p53-mutated TNBC cell lines and, due to the inactivating p53 mutation, this effect is likely independent of an interference with p53-MDM2 interactions, we were interested to know whether the reduction in cancer cell viability occurs independently of cellular p53 expression. To address this important point, we investigated the effect of MDM2 inhibitors on cell viability in the colon carcinoma cells HCT116 *p53^+/+^* and HCT116 *p53^−/−^*. Cells were treated with different concentrations of Idasanutlin, Milademetan, and Nutlin-3a. Additionally, Paclitaxel was used as control, since this drug has been described to induce apoptosis independently of p53 [27]. After performing cell viability assays, inhibitory dose–response curves were generated and IC_50_ values were calculated. All compounds, the MDM2 inhibitors and the chemotherapeutic agent Paclitaxel, reduced the cell viability in a dose-dependent manner in both cell lines regardless of the p53 status (Figure 3). The corresponding IC_50_ values for Idasanutlin, Milademetan, and Nutlin-3a were 4.15 ± 0.31 µM, 6.42 ± 0.84 µM, and 28.03 ± 6.66 µM in HCT116 *p53^+/+^* cells and 5.20 ± 0.25 µM, 8.44 ± 0.67 µM, and 30.59 ± 4.86 µM in HCT116 *p53^−/−^* cells, respectively. For Paclitaxel, the corresponding IC_50_ values were 1.25 ± 0.20 µM and 2.82 ± 0.80 µM in HCT116 *p53^+/+^* and HCT116 *p53^−/−^* cells, respectively. Importantly, for all compounds, no significant difference was observed regarding p53 status.

Taken together, these studies emphasize a mechanism for the anti-tumor activity observed for pharmacological MDM2 inhibition in TNBC independent of p53 function and stresses its therapeutic potential in this breast cancer subtype in the presence of inactivating p53 mutations.

### 2.4. Reduction in Cellular Viability Is Diminished in Non-Malignant Breast Cells Compared to TNBC Cells After Pharmacological Inhibition of MDM2

In addition to the aimed impact on malignant cells, effective drugs also have side effects towards non-malignant cells. This is especially the case for cancer-addressing drugs, since the relevant cellular targets are at least to some extend also present in non-malignant cells. For achieving a therapeutically relevant window, toxicity towards healthy tissues must not exceed that towards cancer cells.

**Figure 4:**
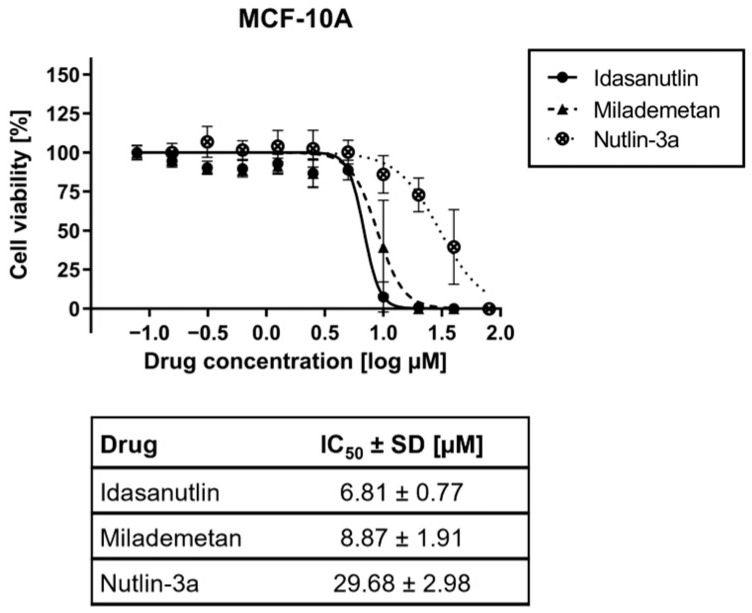
Viability studies of MDM2 inhibitors in non-malignant MCF-10A cells. MCF-10A cells were seeded and incubated for 24 h before treatment. Idasanutlin, Milademetan, or Nutlin-3a were added at the displayed concentrations and incubated for 72 h. Treatment was repeated for another 72 h. Subsequently, cell viability was determined via the CellTiter-Glo assay. The graph shows the mean ± SD n ≥ 3. The table below the graph contains the summarized IC_50_ values ± SD.

MDM2 inhibitors are known to cause on-target side effects in non-malignant cells [13]. To first evaluate the effect of MDM2 inhibition on cellular viability in healthy tissue, we used the non-malignant breast epithelial cell line MCF-10A, which served as a representative of non-malignant breast tissue. When cells were treated with different concentrations of Idasanutlin, Milademetan, or Nutlin-3a, cell viability was reduced in a dose-dependent manner in MCF-10A cells (Figure 4). However, in comparison to the studies performed in TNBC cells, the IC_50_ values of 6.81 ± 0.77 µM for Idasanutlin, 8.87 ± 1.91 µM for Milademetan, and 29.68 ± 2.98 µM for Nutlin-3a were essentially higher in non-malignant MCF-10A cells compared to the TNBC cells. Thus, the IC_50_ value ratio for non-malignant breast cells to malignant TNBC cells was significant and highest for Idasanutlin, with up to a 3.39-fold difference. There was up to a 2.20-fold difference for Milademetan and up to a 1.36-fold difference for Nutlin-3a. Importantly, the cell viability reduction of Idasanutlin and Milademetan was significantly lower than that of Paclitaxel, a genotoxic chemotherapeutic agent currently used for TNBC therapy.

Taken together, our data illustrate a lower reduction of cell viability after pharmacological MDM2 inhibition in non-malignant vs. malignant cells, especially after application of the clinical-stage inhibitors Idasanutlin and Milademetan.

## 3. Discussion

Unraveling tumor-specific vulnerabilities is a key element in the development of novel personalized cancer treatment options that possess reduced toxicity in healthy tissue while showing high therapy efficiency. TNBC is a highly aggressive breast cancer subtype where the targeted therapeutic options are rare and not yet very efficient [3]. This is mainly due to the fact that only a few potential therapeutic targets have been discovered so far, despite intensive basic research.

We here investigated the effect of MDM2 inhibitors on cellular viability in TNBC cell culture models. Determination of cell viability was chosen for apoptosis-inducing drugs since it inversely correlates with cytotoxicity, and IC_50_ values determined in these studies also provide information about the ability to kill or damage TNBC cells. We could prove a high pharmacological potency in several TNBC cell culture models, which was significantly superior in the novel clinical-stage MDM2 inhibitors Idasanutlin and Milademetan compared to the well-characterized non-clinical-stage MDM2 inhibitor Nutlin-3a. Furthermore, we underlined the induction of cell death via apoptosis by detecting an increased caspase-3/7 activity upon cancer cell incubation. Additionally, we unraveled the drug mode of action by MDM2 inhibition to be independent from cellular p53 expression. Finally, MDM2 inhibition illustrated less drug toxicity in non-malignant MCF-10A cells than in TNBC cells. Our studies suggest that targeting MDM2 via specific inhibitors like the novel clinical-stage MDM2 inhibitors Idasanutlin and Milademetan represents a new therapeutic strategy to efficiently compromise TNBC cell viability and to improve current chemotherapy-based TNBC therapy.

Importantly, the significantly higher pharmacological potency of the novel MDM2 inhibitors Idasanutlin and Milademetan, which are currently being tested in clinical trials, compared to the non-clinical-stage MDM2 inhibitor Nutlin-3a aligns with previous studies in p53 wild-type cancer cells and is apparently due to the 15.8- and 14.7-fold higher binding activity for MDM2 compared to Nutlin-3a [18,19,21,26]. Ongoing studies will generate further potent MDM2 inhibitors suitable for clinical applications which, according to our studies here, have high potency to be included in TNBC therapeutic strategies.

While inhibition of MDM2 has widely been shown to be an effective therapeutic approach in p53 wild-type tumors [13,26], little is known about its role in cancers harboring inactivating p53 mutations, including TNBC, especially when tested not only in combination but also as a monotherapy.

One of the most preclinically investigated MDM2 inhibitors is the small molecule inhibitor Nutlin-3a, which reactivates p53 in p53 wild-type cancers and displays significant anti-tumor activity [20]. Interestingly, Lau et al., for the first time, provided evidence that Nutlin-3a apparently also induces apoptosis in p53-mutated HCT116 cancer cells. Comparable to our results, the IC_50_ of 33 µM was in the middle micromolar range. The authors found that apoptosis was not initiated by the inhibition of the protein–protein interaction between MDM2 and mutated p53 but between MDM2 and p73, resulting in the reactivation of p73 [14]. These observations are in line with the reactivation of p73 reportedly effectively inducing apoptosis in TNBC cell lines [28,29,30,31] and the compensation of mutation caused p53’s functional absence via p73 upregulation. Importantly, Adams et al. illustrated that pharmacological degradation of MDM2 by a novel MDM2-specific proteolysis targeting chimera (PROTAC) directly induces apoptosis in TNBC cells via activation of p73 [32].

p73 belongs to the p53 transcription family and shares common features with p53, including apoptosis and cell cycle regulation [33]. Both transcription factors share the same essential amino acid residues of the sequence of the MDM2-binding domain, suggesting the same binding position in the MDM2 pocket [34]. Thus, MDM2, in addition to the interaction with p53 wild-type, likely also binds and inactivates p73, which, in contrast to p53, is not mutated in the vast majority of TNBCs [33]. These data suggest that MDM2 inhibitors might also have a pronounced anti-tumor effect in tumor cells carrying inactivating p53 mutations by reactivating p73 and could successfully function in therapeutic approaches involving personalized treatment for p53-mutated TNBC.

However, to date, it is still unclear how high the pharmacological effectiveness of MDM2 inhibitors in cancers without functional p53 might be. While in our studies we did not observe a significant difference in IC_50_ for all three MDM2 inhibitors between the two HCT116 colon carcinoma cell lines with and without p53 expression, Dudgeon et al. observed a considerable decrease in pharmacological potency for Nutlin-3a in *p53^−/−^* cells [15]. Interestingly, the IC_50_ determined for the MDM2-specific PROTAC developed by Adams et al. was in a comparable range to our results for Idasanutlin and Milademetan (4.0–5.5 µM) in TNBC cells. This anti-tumor activity in the low micromolar range argues for a satisfying anti-tumor potency for Idasanutlin and Milademetan in p53-mutated TNBC [32].

Essentially, the underlying molecular mechanism regarding the anti-tumor activity of MDM2 inhibitors in p53 wild-type tumors and, according to the first studies mentioned, also in p53-mutated/p73 wild-type tumors, is based on cancer cell apoptosis induction [14,35]. The increased caspase-3/7 activity we observed underlines that MDM2 inhibitors, in agreement with other studies [16,17], induce cellular apoptosis also in TNBC cells. In this process, since RNA-seq analysis among different breast cancer cell lines illustrated an intense expression for caspase-3 and a significant expression of caspase-7 in TNBC cell lines, including those we used in our assays, apparently upregulation of caspase-3/7 is less likely to be the major source for increased bioactivity of these enzymes in our assays. Instead, cleavage and activation of these enzymes through other signaling pathways, such as the B-cell lymphoma 2 (BCL-2) family regulated mitochondrial apoptosis signaling pathway, may be responsible [36].

It is important to note that MDM2 inhibitors, based on their apoptosis-inducing activity in TNBC we found in our studies, might also have a high potential to be included in various therapeutic approaches acting synergistically with inhibitors of known pro-oncogenic signaling pathways, including EGF receptor, PIK3CA, AKT, mTOR, Wnt/β-Catenin, NOTCH, TGF, and RAS/RAF/MEK/ERK signaling [4,5,6]. Of note, some of these pathways also have anti-apoptotic functions by acting on BCL-2 associated agonist of cell death protein(BAD) and BCL-2-associated X protein (BAX), upregulating BCL-2, myeloid cellleukemia-1 (MCL-1), survivin, and X-linked inhibitor of apoptosis (XIAP), or downregulating p53 upregulated modulator of apoptosis (PUMA) and BCL-2-interactin mediator of cell death (BIM) [37,38,39,40]. Thus, synergistic targeting using specific inhibitors of the signaling pathways mentioned and MDM2 inhibitors has a high potential to improve personalized therapy for TNBC in the future. Interestingly, the first synergistic studies using Nutlin-3a together with current chemotherapeutics illustrated increased induction of apoptosis in TNBC [16,17].

## 4. Materials and Methods

### 4.1. Cell Cultures

All cell lines were obtained from ATCC (LGC Standards GmbH, Wesel, Deutschland). HCT116 *p53^+/+^* and HCT116 *p53^−/−^* were kindly provided by Dr. Bert Vogelstein (John Hopkins University, Baltimore, MD, USA) and Dr. Thomas G. Hofmann (Institute of Toxicology, University Medical Center Mainz, Mainz, Germany). MDA-MB-231 (ATCC HTB-26, Manassas, VT, USA), MDA-MB-436 (ATCC HTB-130, Manassas, VT, USA), MDA-MB-468 (ATCC HTB-132, Manassas, VT, USA), and NIH-3T3 (ATCC CRL-1658, Manassas, VT, USA) were cultured with Roswell Park Memorial Institute (RPMI) Medium 1640 (Thermo Fisher Scientific Inc., Waltham, MA, USA) supplemented with 10% fetal bovine serum (Thermo Fisher Scientific Inc., Waltham, MA, USA) and 1% penicillin/streptomycin (Thermo Fisher Scientific Inc., Waltham, MA, USA). HCT116 *p53^+/+^* and HCT116 *p53^−/−^* were cultured with McCoy 5a (Thermo Fisher Scientific Inc., Waltham, MA, USA) with 2 mM L-Glutamine (Thermo Fisher Scientific Inc., Waltham, MA, USA), 10% fetal bovine serum, and 1% penicillin/streptomycin. MCF-10A cells (ATCC CRL-10317, Manassas, VT, USA) were cultured with Dulbecco’s modified Eagle medium (DMEM) F12 (Thermo Fisher Scientific Inc., Waltham, MA, USA) supplemented with 5% horse serum (Thermo Fisher Scientific Inc., Waltham, MA, USA), 1% penicillin/streptomycin, epidermal growth factor (EGF) at 20 ng/mL (Miltenyi, Bergisch Gladbach, Germany), hydrocortisone at 0.5 mg/mL (Sigma, #H-0888, St. Louis, MO, USA), cholera toxin at 100 ng/mL (Sigma, #C-8052, St. Louis, MO, USA), and insulin 10 µg/mL (Sigma, #I-1882, St. Louis, MO, USA). All cell lines were incubated in a humidified atmosphere at 37 °C in 5% CO_2_.

### 4.2. Drugs and Reagents

Nutlin-3a was purchased from Cayman Chemicals (Ann Arbor, MI, USA), purity ≥98%. Idasanutlin and Milademetan were obtained from MedChemExpress (Monmouth Junction, NJ, USA), purity ≥98.77%. Nutlin-3a, Idasanutlin, and Milademetan were dissolved in dimethyl sulfoxide (Sigma, #D-2660, St. Louis, MO, USA) to achieve a final concentration of 10 mmol/L in cell culture experiments.

### 4.3. Cell Viability Assay

Cells of all cell lines were seeded at a density of 2.6 × 10^4^ cells per well in 384-well microtiter plates (Greiner Bio-One, Kremsmünster, Austria) and incubated for 24 h at 37 °C before treatment. After removing the culturing medium, serum-reduced medium RPMI 1640 advanced (Thermo Fisher Scientific Inc., Waltham, MA, USA) with 2 mM L-Glutamine, and 100 nM dexamethasone (Sigma, #D-4902, St. Louis, MO, USA) was used for treatment. For MCF-10A cells, DMEM F12 supplemented with 2% horse serum (Thermo Fisher Scientific Inc., Waltham, MA, USA), hydrocortisone (Sigma, #C-0888, St. Louis, MO, USA) at 0.5 mg/mL, cholera toxin (Sigma, #C-8052, St. Louis, MO, USA) at 100 ng/mL, and insulin (Sigma, #I-1882, St. Louis, MO, USA) at 10 µg/mL was used as the assay medium. Compounds were added at the mentioned concentrations ranging from 0.08 µM to 80 µM and incubated for 72 h and repeated for another 72 h. Subsequently, cell viability was evaluated using the CellTiter-Glo Luminescent Cell Viability Assay (Promega, Madison, WI, USA) according to the manufacturer’s instructions. All measurements were performed using the Spark multimode microplate reader (Tecan Group AG, Männedorf, Switzerland).

### 4.4. Caspase-3/7 Activity Assay

MDA-MB-231 cells were seeded at a density of 2.6 × 10^4^ cells per well in 384-well microtiter plates (Greiner Bio-One, Kremsmünster, Austria) and incubated for 24 h at 37 °C before treatment. After removing the culturing medium, serum-reduced medium RPMI 1640 advanced (Thermo Fisher Scientific Inc., MA, USA) with 2 mM L-Glutamine (Thermo Fisher Scientific Inc., MA, USA), and 100 nM dexamethasone (Sigma, #D-4902, St. Louis, MO, USA) was used for treatment. Compounds were added at the mentioned concentrations and incubated for 24 h. Subsequently, caspase-3/7 activity was evaluated using the Caspase-Glo 3/7 Assay System (Promega, Madison, WI, USA) according to the manufacturer’s instructions. All measurements were performed using the Spark multimode microplate reader (Tecan Group AG, Männedorf, Switzerland).

### 4.5. Statistical Analysis

All experiments were performed at least three times. The results are expressed as the mean ± standard deviation (SD) of the mean. Student’s *t*-test or one-way Analysis of Variance (ANOVA) with Dunnett’s or Tukey’s post hoc tests were performed. All analyses were conducted using GraphPad PRISM Version 8.0.2. A probability of error of *p* < 0.05 was considered statistically significant.

## 5. Conclusions

Taken together, using current clinical-stage MDM2 inhibitors, we here report pharmacological MDM2 inhibition to potently reduce cellular viability in TNBC. We observed this significant anti-tumoral effect in different p53-mutated TNBC cell lines and assume that the underlying molecular mechanism involves induction of apoptosis independent of functional p53 but presumably involving pathways associated with p73. Importantly, the drug effect on cellular viability in non-malignant cells was reduced compared to TNBC cells.

These data suggest that pharmacological MDM2 inhibition has a high potential to serve as a basis for the further development of personalized TNBC therapies and might offer novel therapeutic options in targeted TNBC treatments in the future.

### Limitations

Although our cellular studies were performed in three molecularly and phenotypically different TNBC cell lines all possessing an inactivating p53 mutation [25], efficiency investigations in primary TNBC cells might differ from the established cell culture models. Therefore, further studies in patient-derived organoids are required to exclude reduced therapeutic effects in patient cancer cells. Furthermore, the complexity of a whole organism with the interplay of different malignant and non-malignant cells including the tumor microenvironment could not be addressed in our studies and must be investigated in future orthotopic PDX animal models. Additionally, given the likelihood of a multimodal therapeutic approach for highly aggressive cancers such as TNBC, additional synergistic studies with current TNBC therapeutics will be helpful to evaluate clinical-stage MDM2 inhibitors in adjuvant therapies of TNBC, which has not scientifically been addressed yet.

## Data Availability

The datasets generated during and/or analyzed during the current study are not publicly available due to a solely in-house electronic data archiving but are available from the corresponding author on reasonable request.

## Supplementary Materials

The supporting information can be downloaded at: https://www.mdpi.com/article/10.3390/ijms26031078/s1.
