# Supplementary material for: Pharmacological Inhibition of MDM2 Induces Apoptosis in p53-Mutated Triple-Negative Breast Cancer
Source: Int J Mol Sci. 2025 Jan 26;26(3):1078. doi: 10.3390/ijms26031078 (PMC11817430; doi:10.3390/ijms26031078)
Supplement: Supplementary file 1 [file ijms-26-01078-s001.zip › Supplementary Data On et al.pdf]

Supplementary Data

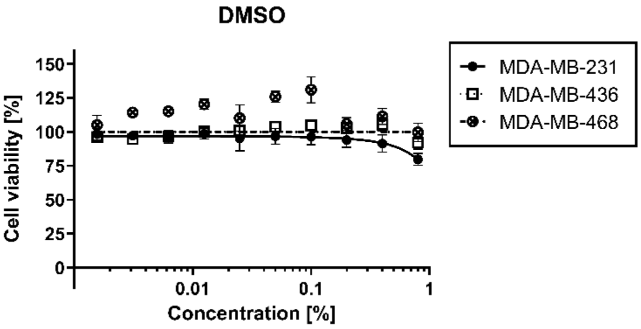

Figure S1: Analysis of cell viability in TNBC cell lines after incubation with the vehicle control dimethyl sulfoxide (DMSO). MDA-MB-231, MDA-MB-436 or MDA-MB-468 were seeded and incubated for 24 hours before treatment. DMSO as vehicle control was added at displayed concentrations and incubated for 72 hours. Treatment was repeated for another 72 hours. Subsequently, cell viability was determined by CellTiter -Glo assay. The graph shows the mean ± SD n≥3.
